# Simple Detection of the IS*6110* Sequence of *Mycobacterium tuberculosis* Complex in Sputum, Based on PCR with Graphene Oxide

**DOI:** 10.1371/journal.pone.0136954

**Published:** 2015-08-31

**Authors:** Sang-Hyun Hwang, Dong-Eun Kim, Heungsup Sung, Byeong-Min Park, Mi-Jeong Cho, Ok-Jin Yoon, Do-Hoon Lee

**Affiliations:** 1 Department of Laboratory Medicine, Center for Diagnostic Oncology, Research Institute and Hospital, National Cancer Center, Goyang-si, Gyeinggi-do, 410–769, Republic of Korea; 2 Hematologic Malignancy Branch, Research Institute and Hospital, National Cancer Center, Goyang-si, Gyeinggi-do, 410–769, Republic of Korea; 3 Department of Bioscience and Biotechnology, Konkuk University, Seoul, 143–701, Republic of Korea; 4 Department of Laboratory Medicine, University of Ulsan College of Medicine and Asan Medical Center, 388–1 Pungnap-dong, Songpa-gu, Seoul, 138–736, Republic of Korea; Institut de Pharmacologie et de Biologie Structurale, FRANCE

## Abstract

Graphene oxide (GO) has proven to be a satisfactory DNA-sensor platform for applications in enzyme-free signal amplification, fluorescence-based amplification, and nanoparticle-based platforms because of its excellent electrical, thermal, and optical properties. In this study, we designed a novel platform for the fluorescence detection of biomolecules, using a fluorescent dye-labeled primer and GO. We applied this system for the detection of the IS*6110* insertion sequence of the *Mycobacterium tuberculosis complex* (MTB) and evaluated its feasibility for use in molecular diagnostics. Fifty-four sputum specimens were collected at our institution from October 2010 to March 2012. To detect MTB in the samples, we performed PCR amplification of the IS*6110* DNA sequence using FAM-labeled primers, after which the PCR amplicon was incubated with GO and the fluorescence was measured. The results were compared with those obtained by conventional real-time quantitative PCR (RQ-PCR). The fluorescence intensity observed increased in a concentration-dependent manner with the FAM-labeled IS*6110* amplicon. The results of the PCR-GO system for detecting IS*6110* DNA were in good agreement with those obtained with conventional RQ-PCR (kappa statistic = 0.925). The PCR-GO system detected MTB DNA in 23 of 25 RQ-PCR-positive sputum samples (92.0%; 95% CI, 75.0–98.0%), but not in 29 of 29 RQ-PCR-negative sputum samples (100%; 95% CI, 88.1–100.0%). These results indicate the utility of the PCR-GO system in molecular diagnostics.

## Introduction

Graphene is a one-atom-thick planar sheet of sp2-bonded carbon atoms that forms a honeycomb crystal lattice [1]. Graphene is an attractive nanomaterial because of its unique optical, physical, and electrochemical properties, which make it promising for biosensing applications [2,3] and nucleic acid detection [4,5]. Optically, graphene shows light absorption from the ultraviolet to the near-infrared regions. In addition, graphene is an efficient fluorescence quencher that is useful in the optical-based detection of biomolecules [6–9]. Graphene oxide (GO) is a water-soluble derivative of graphene that preferentially associates with single-stranded (ss) nucleic acids by π-π stacking interactions, with a lower affinity for double-stranded (ds) nucleic acids [3,10]. Therefore, GO could potentially be used as a fluorescence quencher for the detection of DNA sequences instead of conventional fluorescent probes, such as TaqMan or Molecular Beacon probes. Using GO as a quencher may be advantageous in that it more efficiently quenches fluorescence than other commonly used quenchers. In addition, GO can serve as a universal quencher, in contrast to conventional fluorescent probes such as TaqMan and Molecular Beacon probes, which are FRET-based donor-acceptor (quencher) dye pairs that must be prepared for each target sequence being detected [6].

Tuberculosis (TB) is caused by gram-positive bacteria that are members of the *Mycobacterium tuberculosis* (MTB) complex, including such human pathogens as *M*. *tuberculosis* and *M*. *africanum*. TB poses a serious global health threat, with 9 million new cases and 1.5 million deaths reported worldwide in 2013 [11]. Multidrug-resistant (MDR) TB strains, with which patients fail to respond to the first-line drugs isoniazid and rifampin, are spreading rapidly and threaten to undermine the control of TB [12]. Reliable, early, and sensitive diagnosis of the MTB complex is required for the effective treatment of patients and disease control [12]. The sensitivity of sputum-smear microscopy is low, and the culture method, which is generally used to confirm diagnoses made by microscopy, typically takes several weeks due to the slow growth of MTB, although the use of liquid culture media has reduced the time required for MTB detection [13]. Nucleic-acid amplification tests based on methods such as real-time quantitative PCR (RQ-PCR) or isothermal amplification are currently the most promising methods for clinical diagnostics [14–17].

In this study, we developed a simple, separation-free method for MTB DNA detection based on the quenching of GO-bound fluorophores. During the amplification step, a 6-carboxyfluorescein (FAM)-labeled primer is incorporated into the amplicon. In the absence of the target, it remains free in solution. Once GO is added, the single-stranded primer adsorbs to GO, resulting in quenching of the FAM fluorescence. Adsorption of the double-stranded amplicon to GO is less favorable; thus, fluorescence is detected in the presence of the target amplicon. A low-cost fluorometer was used for detection purposes. In recent studies, non-amplified single-stranded RNA sequences such as microRNAs were captured and detected using GO [18]. However, we first amplified the target DNA by PCR and then measured the fluorescence from dye-labeled primers that were incorporated into the target amplicons, resulting in desorption from GO and fluorescence signaling. In addition, we evaluated the feasibility of our PCR-GO method for detecting MTB and compared its performance with that of a commercial RQ-PCR test.

## Materials and Methods

### Sample collection and DNA extraction

We used 54 sputum specimens that had been stored after completing a previous study for MTB detection [19]. Out of the 54 specimens tested, 46.3% (25/54) were MTB-positive. The sputum samples (1 mL) were inactivated by heating at 80°C for 60 min, and 200 μL of each sample was used for DNA extraction with the QIAamp DNA Stool Mini Kit (QIAGEN, Valencia, CA, USA), according to the manufacturer’s instructions.

### IS*6110* PCR

Gene-specific primers targeting the IS*6110* sequence (GenBank Accession No. X52471) were designed with the Primer3 program [20]. The nucleotide sequences of the forward and reverse primers were 5′-FAM-GCCTACGTGGCCTTTGTCAC-3′ and 5′-C3-GTCCAGATGGCTTGCTCGAT-3′, respectively, where C3 is a 3-carbon spacer. The amplicon size was 113 bp. Primers were synthesized by Integrated DNA Technologies (Coralville, Iowa, USA).

Amplification was performed using the HotStarTaq Plus Master Mix Kit (QIAGEN), as described previously [19]. Reaction mixtures contained 0.25 μM of each dNTP, 0.4 μM forward primer, 0.3 μM reverse primer, and 5 μL of sample DNA. A GeneAmp PCR System 9700 thermal cycler (Applied Biosystems, Foster City, USA) was used for DNA amplification. The thermal cycling conditions used were as follows: 5 min at 95°C; 40 cycles at 94°C for 30 s, 65°C for 30 s, and 72°C for 30 s; and 72°C for 10 min. All experiments were performed in duplicate.

For comparison purposes, conventional RQ-PCR was performed using the AdvanSure TB/NTM Real Time PCR Kit (LG Lifesciences, Seoul, Korea) on the ABI Prism 7000 Sequence Detection System (Applied Biosystems), as described previously [19].

For serial dilution sensitivity testing, we generated plasmids containing the IS*6110* DNA sequence (p-IS*6110*) as described previously [19], and a 10-fold dilution series (10^1^–10^6^ copies/ μL) was prepared.

### Detection of amplification using GO as a fluorescence quencher

**Fig 1:**
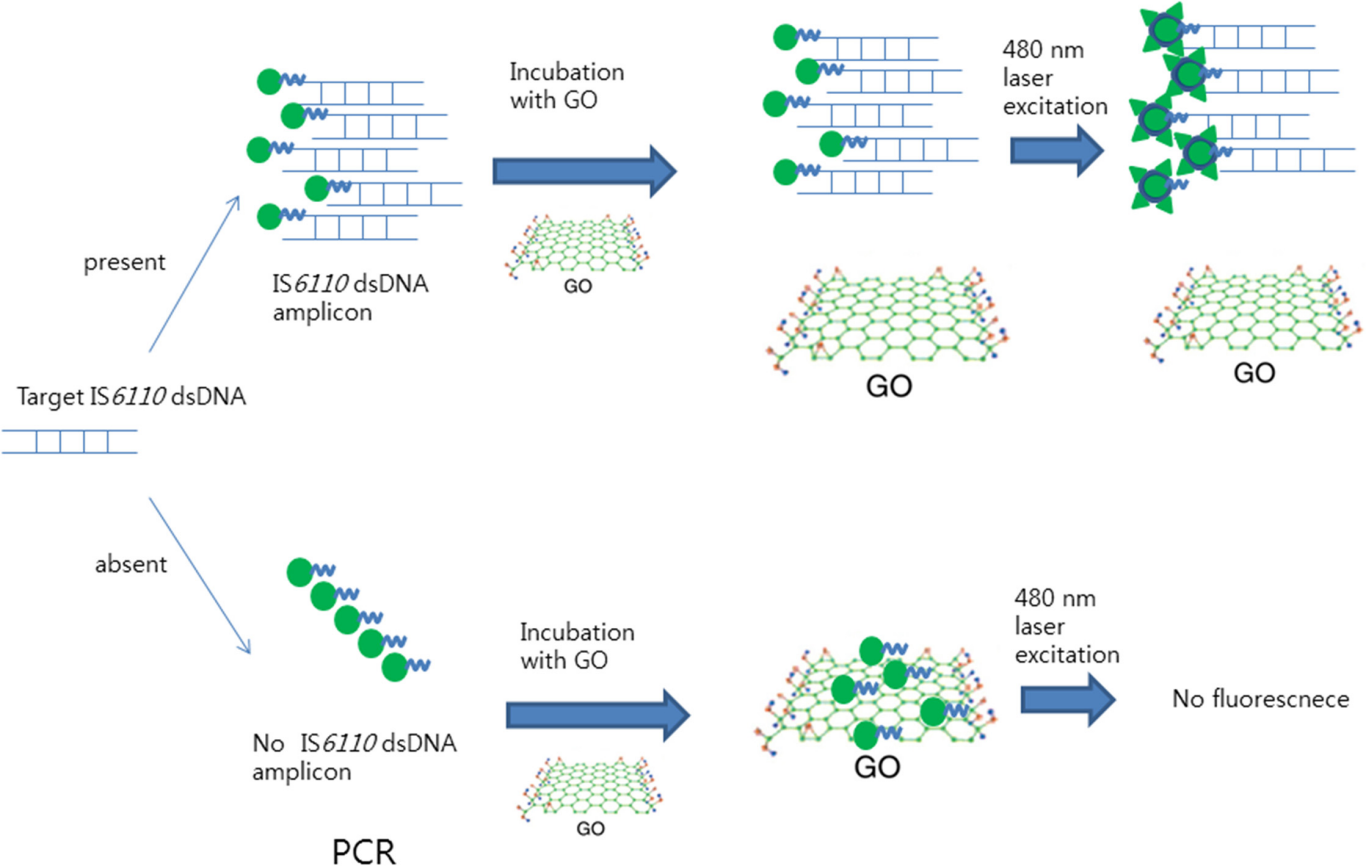
Illustration describing the GO-based sensing system for homogenous detection of the IS*6110* DNA amplicon. In the presence of the IS*6110* DNA amplicon, the fluorescent dye-labeled primers are incorporated into the amplicon causing the primers to separate from GO, leading to increased fluorescence (upper part of the figure). In the absence of the target DNA, the fluorescent dye-labeled primers remain adsorbed to the GO layer, resulting in fluorescence quenching (lower part of the figure).

The principle of the GO-based amplification detection system is illustrated in Fig 1. Briefly, after PCR amplification, 20 μL of FAM-labeled PCR amplicon was mixed with 20 μL of 1 mg/mL stock of GO (Graphene Square, Seoul, South Korea) and 60 uL of distilled water for a final GO concentration of 0.2 mg/mL, and the mixture was incubated for 15 min at room temperature. The fluorescence intensities of the samples were measured with the Qubit 2.0 Fluorometer (Invitrogen, Eugene, OR, USA), using the blue LED (max ~470 nm) excitation light source. The fluorescent signals were recorded as relative fluorescent units (RFUs) using a green fluorescence emission filter (510–580 nm), and the RFU (sample)/RFU (control) ratios were calculated.

## Results

### The fluorescent signal detected using the PCR-GO system is proportional to the amplicon concentration

**Fig 2:**
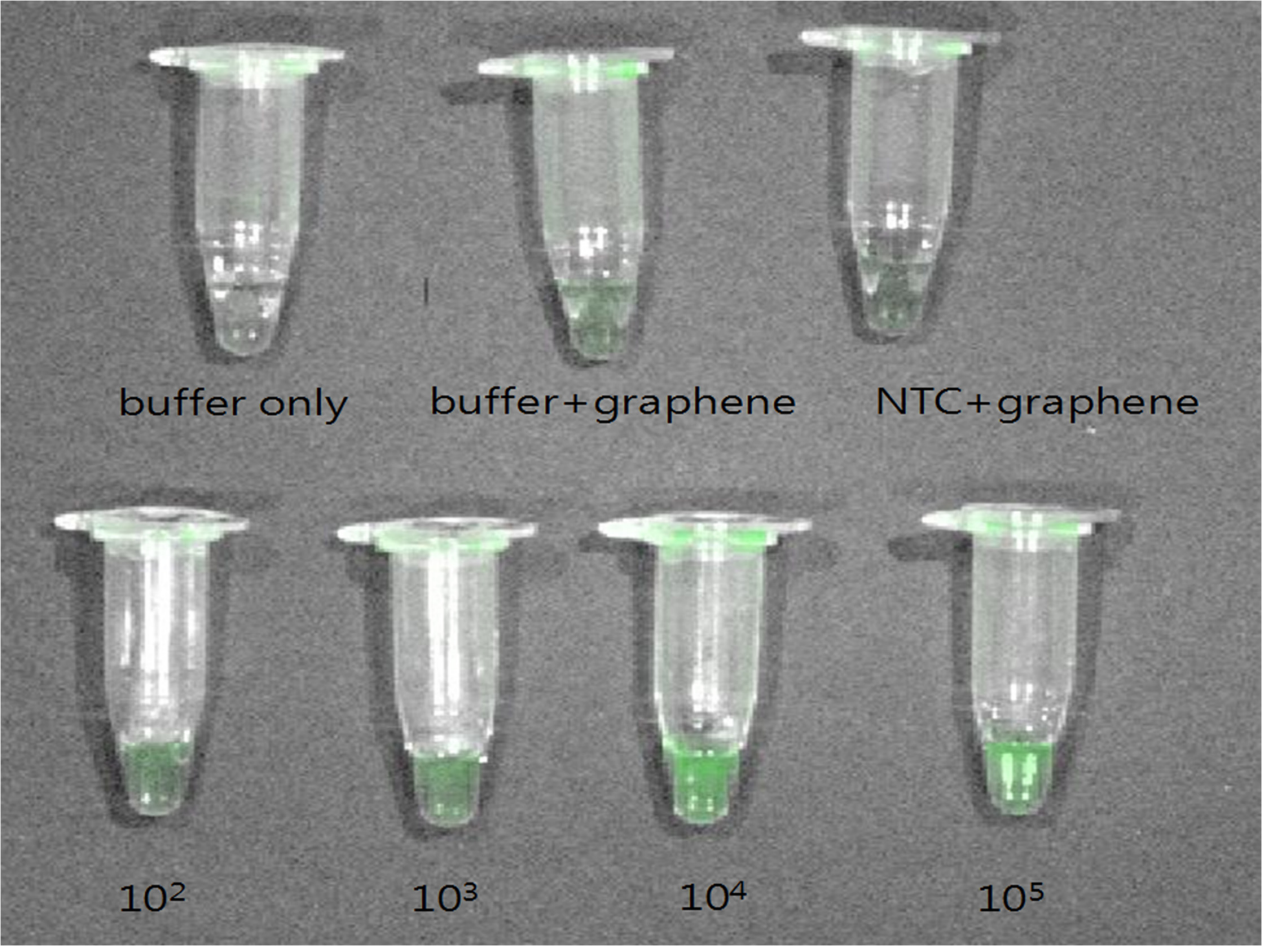
Fluorescence signaling after PCR-GO is proportional to the amplicon concentration. Fluorescence in tubes containing 0 (NTC) to 10^5^ copies/reaction of a plasmid encoding the IS*6110* sequence is shown. Twenty microliters of a 1 mg/mL stock of GO was added at a final concentration 0.2 mg/mL. The fluorescence images were obtained using the IVIS Lumina Series Ⅲ imaging system. NTC, no-template control.

As shown in Fig 2, the fluorescence intensity displayed gradual increases with increasing concentrations of fluorescent dye-labeled amplicons.

### Sensitivity assay of PCR-GO detection

**Fig 3:**
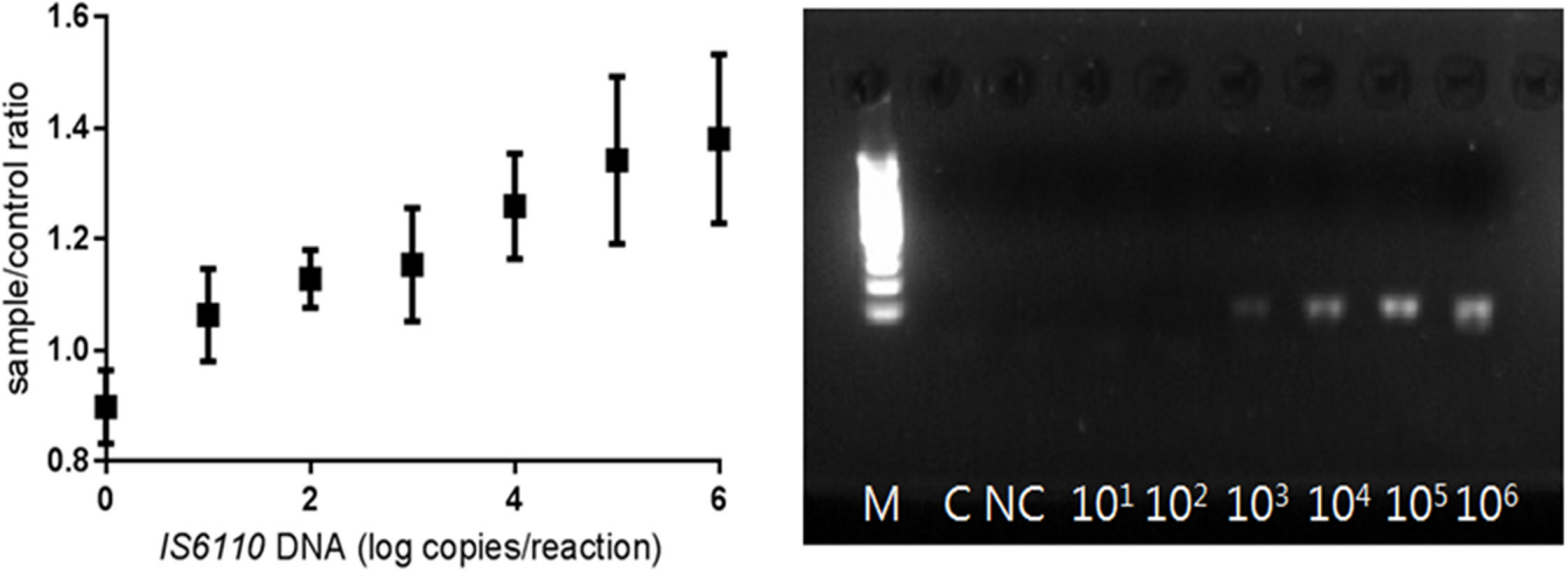
Detection sensitivity of the PCR-GO system. Left, The RFU ratios (sample/control) increased in proportion to the concentration of p-IS*6110* used for PCR amplification (10^1^–10^6^ copies/reaction). Negative group (no template control) was significantly different from positive groups (10^1^, 10^2^, 10^3^, 10^4^, 10^5^, 10^6^ copies/reaction) (Tukey-Kramer multiple-comparison test, *p* < 0.05). 10 replicates were performed at each concentration. Right, Gel electrophoresis of the 113-bp amplicons after PCR amplification using 10^1^–10^6^ copies of the p-IS*6110* plasmid per reaction, as indicated. Right, C, blank; NC, no-template control.

To determine the detection sensitivity of the PCR-GO method, the p-IS*6110* plasmid was serially diluted and amplified using a primer pair, one of which was FAM-labeled. Subsequently, the PCR amplicons were incubated with GO, after which the fluorescence intensity was measured. The fluorescence intensity at 543 nm and the RFU ratios increased proportionally to the increase in p-IS*6110* amplicon concentration (Fig 3).

### Performance of the PCR-GO system with clinical samples

Finally, we evaluated the performance of the PCR-GO system for detecting IS*6110* DNA in clinical samples. The results were in good agreement with those obtained using a conventional RQ-PCR kit (kappa statistic = 0.925). The PCR-GO system detected MTB DNA in 23 out of 25 RQ-PCR-positive samples (92.0%; 95% CI: 75.0–98.0%) but not in 29 out of 29 RQ-PCR-negative samples (100%; 95% CI: 88.1–100.0%).

## Discussion

In this study, we explored the feasibility of using GO as a DNA biosensor for detecting the IS*6110* sequence of MTB and prototyped a PCR-GO detection system for potential applications in molecular diagnostics. We evaluated the performance of the PCR-GO system in comparison to the commercial AdvanSure RQ-PCR kit that is approved for *in vitro* diagnostics by the Korean Food and Drug Administration. In a previous study, we used this kit to detect TB in 129 culture–positive TB specimens. We found that 82 out of 82 AFB-stain-positive specimens (100%) and 35 of 47 (74.5%) AFB-stain-negative specimens were detected using this kit, indicating a sensitivity of 90.7% and a specificity of 100% [21]. To the best of our knowledge, no previous studies have been performed to compare the PCR-GO method with current clinical molecular-detection techniques. The PCR-GO results showed strong agreement with the results obtained using conventional RQ-PCR (96.3% agreement; kappa = 0.925). The PCR-GO-based fluorometric assay is cost-effective and does not need spectrally matched reporter and quencher dyes, as used in TaqMan assays.

Some MTB strains contain do not encode the target DNA IS*6110* sequence in their genomes, and this assay would not be suitable in such cases [22].

One of advantages of GO is that it more efficiently quenches the fluorescence of common fluors. GO can also serve as an effective universal quencher, without the need for designing new dual-labeled probes for each target, as is done with conventional fluorescent probes such as TaqMan and Molecular Beacon probes [6–9]. Moreover, primers labeled with different fluors in combination with GO as quencher can be used for multiplexing, in contrast to detection via intercalating dyes such as SYBR green that do not enable multiplexing. However, GO could be used for multiplexing if the primers were labeled with different fluors. In addition, the cost per sample of PCR-GO is less expensive. The cost per sample of a FAM-labeled primer was ~$0.2 and that of GO was ~$0.02. In contrast, the cost per sample of dual-labeled probes including a quencher dye was ~$0.7 in this study.

The appropriate design of DNA probes and optimization of GO concentrations was important for improving the specificity of detection of IS*6110* DNA based on the GO platform. DNA absorption/desorption from GO is critically affected by salts, pH, temperature, and organic solvents such as ethanol [9,23]. The amplicon and primer/probe sizes have also been shown to affect DNA absorption/desorption from GO [9]. In agreement with previous findings, we observed that fluorescent signal intensities varied according to amplicon sizes (data not shown). The optimal GO concentration for quenching fluorescence emission was found to be 0.2 mg/mL (S1 Fig). Similar concentrations (0.25–0.4 mg/mL) of GO were used in other studies [24,25].

One drawback of our PCR-GO system is that primer-dimers can produce false positive signals. Another disadvantage is that the end point-detection format requires opening of the tube containing amplicon to add the GO. This introduces significant risk of amplicon carryover, and subsequent false positives. Real-time detection formats provide a key advantage in that tubes containing amplicon do not have to be opened up again. However, lab-on-a-chip technologies including microfluidics could add amplicons to the GO in closed system in the future.

However, unresolved issues and technical problems remain, such as optimizing the probe size, as well as the surface modification and structure of GO, which could enhance the performance of the GO-based DNA detection platform. Therefore, further studies should be performed to improve the sensitivity of the PCR-GO system.

The PCR-GO assay was developed using the Qubit 2.0 fluorometer, which is a small, low-cost, easy-to-use analytical instrument for DNA, RNA, and protein quantitation. This fluorometer has 2 light sources (blue and red LEDs), 2 excitation filters (blue: 430–495 nm and red: 600–645 nm), and 2 emission filters (green: 510–580 nm and red: 665–720 nm). FAM has an excitation/emission peak at 495/520 nm. Therefore, we could detect the fluorescence signal from the FAM-labeled primers with the Qubit 2.0 fluorometer.

In conclusion, we developed a novel and simple PCR-GO-based system and demonstrated its application in detecting MTB. In this study, we showed that the performance of our detection system using a simple fluorometer was comparable to that of conventional RQ-PCR. Improvements and optimization of the PCR-GO system are challenges that merit further attention. In the future, biosensors, lab-on-chip technologies, and microfluidics systems are just some of the technologies in which GO-based analysis can be integrated to generate highly sensitive *in vitro* diagnostic devices.

## Supporting Information

S1 Fig Optimization of the GO concentration for FAM-fluorescence quenching. (DOCX)
