# Supplementary material for: Simple Detection of the IS6110 Sequence of Mycobacterium tuberculosis Complex in Sputum, Based on PCR with Graphene Oxide
Source: PLoS One. 2015 Aug 31;10(8):e0136954. doi: 10.1371/journal.pone.0136954 (PMC4556190; doi:10.1371/journal.pone.0136954)
Supplement: S1 Fig — (DOCX) [file pone.0136954.s001.docx]

**S1 Figure. Optimization of the GO concentration for FAM-fluorescence quenching**

S1 Figure. The effect of the GO concentration on FAM-fluorescence quenching. Fluorescence quenching increased in direct proportion to increasing GO concentrations. The minimum concentration of GO required for complete quenching was at a final GO concentration of 0.2 mg/mL. The relative fluorescence units (RFUs) were measured using a Qubit 2.0 fluorometer. Fluorescence images were obtained using the IVIS Lumina Series Ⅲimaging system (PerkinElmer, Waltham, MA, USA).
